# Loss of Lkb1 in CD11c^+^ myeloid cells protects mice from diet-induced obesity while enhancing glucose intolerance and IL-17/IFN-γ imbalance

**DOI:** 10.1007/s00018-023-04707-w

**Published:** 2023-02-13

**Authors:** Yunyan Sun, Bing Wang, Qianwen Hu, Haixiao Zhang, Xun Lai, Tier Wang, Chunxiao Zhao, Jiali Wang, Xi Zhang, Qing Niu, Baolin He, Erlie Jiang, Mingxia Shi, Xiaoming Feng, Yuechen Luo

**Affiliations:** 1 State Key Laboratory of Experimental Hematology, National Clinical Research Center for Blood Diseases, Haihe Laboratory of Cell Ecosystem, Institute of Hematology & Blood Diseases Hospital, Chinese Academy of Medical Science & Peking Union Medical College, Tianjin, 300020 China; 2 Tianjin Institutes of Health Science, Tianjin, 301600 China; 3 Department of Hematology, Hematology Research Center of Yunnan Province, The First Affiliated Hospital of Kunming Medical University, Kunming, China; 4 Department of Hematology, Yunnan Cancer Hospital, The Third Affiliated Hospital of Kunming Medical University, Yunnan Cancer Center, Kunming, China

**Keywords:** Visceral adipose tissue, Dendritic cells, Macrophages, T cells, IL-17A, IFN-γ, Liver kinase B1, High-fat-diet-induced obesity

## Abstract

Adipose tissue CD11c^+^ myeloid cell is an independent risk factor associated with obesity and metabolic disorders. However, the underlying molecular basis remains elusive. Here, we demonstrated that liver kinase B1 (Lkb1), a key bioenergetic sensor, is involved in CD11c^+^ cell-mediated immune responses in diet-induced obesity. Loss of *Lkb1* in CD11c^+^ cells results in obesity resistance but lower glucose tolerance, which accompanies tissue-specific immune abnormalities. The accumulation and CD80’s expression of Lkb1 deficient adipose-tissue specific dendritic cells but not macrophages is restrained. Additionally, the balance of IL-17A and IFN-γ remarkably tips towards the latter in fat T cells and CD11c^−^ macrophages. Mechanistically, IFN-γ promotes apoptosis of preadipocytes and inhibits their adipogenesis while IL-17A promotes the adipogenesis in vitro, which might account in part for the fat gain resistant phenotype. In summary, these findings reveal that Lkb1 is essential for fat CD11c^+^ dendritic cells responding to HFD exposure and provides new insights into the IL-17A/IFN-γ balance in HFD-induced obesity.

**Supplementary Information:**

The online version contains supplementary material available at 10.1007/s00018-023-04707-w.

## Introduction

The immune and metabolic systems are critical for species survival and share many underpinnings [1]. Obesity, a representative metabolic immune disorder in adipose tissue, has become a serious public concern over the past few decades [2–4]. Adipose tissue plays a systemically metabolic role and is also an endocrine immune organ expressing high levels of adiponectin to preserve systemic insulin sensitivity and adipose immune balance [5]. However, continuous overnutrition can lead to pathological expansion of adipocytes. Excess adiposity makes the enlarged adipocytes more susceptible to endoplasmic reticulum stress and death, resulting in local hypoxia and ectopic lipids. These damage-associated molecular patterns and metabolic signals recruit dendritic cells, macrophages, and other immune cells and initiate proinflammatory responses such as TNF-α secretion, which further disrupts the metabolic function of adipocytes. Such a metabolic-immune vicious cycle alters systemic glucose and lipid metabolism and insulin action, leading to metabolic diseases, including insulin resistance and type 2 diabetes [1, 6–8].

Various immune cells and immune cytokines compose the adipose tissue immune environment. Macrophages, dendritic cells (DC), CD4^+^ Th1/17 cells, CD8^+^ cytotoxic T cells, B cells, and mast cells are recognized as pro-inflammatory cells, which are responsible for the metabolic pathogenesis of obesity. CD4^+^Foxp3^+^ regulatory T cells (T_reg_), Th2 cells, and eosinophils are recognized as protective cells that maintain a healthy status and insulin sensitivity [9–11]. Among these immune populations, CD11c^+^ cells are considered a therapeutic target for obesity-associated metabolic disorders because ablation of this population could alleviate the obese-induced insulin sensitivity [12–16]. Fat CD11c^+^ cells are composed of adipose tissue macrophages (ATM) and dendritic cells (ATDC), which could be clearly distinguished with CD64 and CD11c in CD45^+^cells [14, 17]. There is another well-recognized gating strategy as “CD45^+^MHCII^+^CD11c^hi^” to identify ATDC [13].

T_reg_-mediated immune suppression plays a central role in minimizing harmful immune responses and maintaining peripheral tolerance [18]. Visceral adipose tissue (VAT) resident T_regs_ are more abundant than lymphoid T_regs_ and exhibit sex-specificity; they are derived from thymic T_regs_ during the early weeks after birth. Their expansion is dependent on antigens presented by DCs and cytokines such as IL33. It could also be recruited via the CCL2-CCR2 axis. Notably, VAT T_regs_ are specifically and strikingly reduced in obese mice with metabolic disorders [19–22]. Surrogate agonist peptides that cause VAT T_regs_ expansion could effectively protect the AT from inflammation and improve metabolic indices [23]. These results indicate that VAT T_regs_ are also promising therapeutic targets to restrain the metabolic-immune vicious cycle and improve metabolic disease.

Liver kinase B1 (Lkb1), as a serine-threonine kinase, is defective in cancer-susceptible Peutz–Jeghers syndrome [24]. Our previous study revealed that Lkb1 stabilizes the expression of Foxp3 and maintains T_regs_ lineage stability [25]. In addition, *Lkb1* deletion in CD11c^+^ dendritic cells can strongly promote lymphoid T_regs_ expansion in contact manner [26, 27]. It has also been reported that Lkb1 coordinates metabolic and immune quiescence of DCs to enforce antitumor immunity [28]. However, the specific role of Lkb1 in CD11c^+^ myeloid cells in diet-induced obesity and VAT T_regs_ has not yet been studied.

In the present study, we generated CD11c^Cre^ Lkb1^f/f^ mice to elucidate the role Lkb1 of in CD11c^+^ cells under high-fat diet exposure. We found that *Lkb1* deletion in CD11c^+^ cells leads to an obesity-resistant phenotype in a high-fat diet (HFD)-induced obesity (DIO) model. We dissected the differences in adipose tissue immune profile between CD11c^Cre^ Lkb1^f/f^ mice and Lkb1^f/f^ mice. The results showed that, the lost of Lkb1 in CD11c^+^ cells caused several changes regarding immune factors. 1. Reduction in ATDC’s accumulation and CD80 expression; 2. The advantage of VAT T_regs_ in CD11c^Cre^ Lkb1^f/f^ mice narrowed in DIO model; 3. The effector-cytokine balance of IL-17A and IFN-γ tips towards the latter in both VAT-T cells and ATMs. Mechanistically, the addition of IFN-γ inhibits the differentiation of preadipocytes into adipocytes and increases apoptosis while IL-17A promotes the adipogenesis ex vivo. Thus, these findings reveal a dominant role of CD11c^+^ dendritic cells’ Lkb1 in shaping the immune microenvironment of adipose tissue and provide new insights into the IL-17A/IFN-γ balance in HFD-induced obesity.

## Material and methods

### Mice and diet

All animals were raised in specific pathogen-free barrier (SPF) facilities. We used these animals following the protocol approved by the Institutional Animal Care and User Committee at the Institute of Hematology, Chinese Academy of Medical Sciences. CD11c^Cre^ and Lkb1^f/f^ mice purchased from Jackson Laboratories (Bar Harbor, ME, USA) were backcrossed with C57BL/6 mice for at least seven generations. Lkb1^f/f^ mice were hybridized with CD11c^Cre^ ones to produce CD11c^Cre^Lkb1^f/f^ mice. In control and obesity group, male mice were fed with an NFD (product data-D12450B, 3.85 kcal/g, 20% protein, 10% fat, 70% carbohydrates) [29] and an HFD (research diets D12492, 5.24 kcal/g, 20% protein, 60% fat, 20% carbohydrates), respectively, from 6 weeks of age [15], for 16 weeks. We housed the different genotypic littermates in one cage for the same experiments.

### Metabolic evaluation

From the beginning of HFD feeding, the body weight of the experimental mice was measured weekly, and fasting blood glucose was measured every four weeks after 12 h of fasting during the night (around 8:00 p.m.–8:00 a.m.).

### Intraperitoneal glucose tolerance test (IPGTT)

The mice were fasted for 6 h and then intraperitoneal injection with glucose (1.0 g/kg), and the blood glucose concentration (mmol/L) was measured with a glucose meter at 0, 15, 30, 60, and 120 min after injection [15, 30]. The area under the curve (AUC) was obtained by calculating the space between the x-axis and a given curve using GraphPad Prism software (GraphPad Software, version 7.0a).

### Intraperitoneal insulin tolerance test (IPITT) and insulin secretion

The mice were fasted for 6 h and then intraperitoneal injection with insulin (1.0 U/kg), and the blood glucose concentration (mmol/L) was measured with a glucose meter at 0, 15, 30, 60, 90, and 120 min after injection [15, 30]. Blood insulin was tested during the glucose tolerance test [31].

### Fat-associated stromal vascular fraction (SVF) cell isolation

Epididymal fat pads (VAT) and subcutaneous adipose tissue (SAT) around the lower limbs of male CD11c^Cre^Lkb1^f/f^ and Lkb1^f/f^ mice fed an NFD or HFD for 16–20 weeks were surgically dissected and weighed. The AT was chopped with scissors and digested by rotation with 1 mg/mL collagenase at 37 °C (Sigma-Aldrich, Germany) for 30 min. Digestion was terminated by Dulbecco's modified Eagle's medium (DMEM/F-12) (Gibco, USA) with 2% fetal bovine serum (FBS), followed by centrifugation at 1600 rpm for 5 min. The upper layer of mature adipocytes was discarded, and the precipitated interstitial vascular fraction was further purified by density gradient centrifugation to obtain the immune cells.

### Adipocyte measurement

The AT obtained by dissection was fixed in 10% formalin. Sections were then stained with hematoxylin–eosin staining, observed with a microscope (100 ×), and analyzed with ImageJ software [15]. Three regions were randomly collected, and the average cell area was determined by calculating the number of cells and the total size in the region.

### Flow cytometry

Single-cell suspensions were prepared from the spleen and VAT and stained with PE-MHC-II (107613, Biolegend), APC-cy7-CD11c (117323, Biolegend), FITC-CD64 (139315, Biolegend), Percp-cy5.5-CD45.2 (109827, Biolegend), APC-CD45 (147707, Biolegend), FITC-CD4 (100405, Biolegend), APC-CD8 (126613, Biolegend), PE-cy7-CD62L (104417, Biolegend), Percp-cy5.5-CD44 (103031, Biolegend), APC-cy7-FVD, or Pacific blue-DAPI. Intracellular staining was performed using PE-Foxp3 (12-5773-82, eBioscience) and antibodies against cytokines, including BV421-CD8, PE-cy7-IFN-γ (505825, Biolegend), Percp-cy5.5-IL-17A (506919, Biolegend), and APC-IL-4 (504105, Biolegend). Cells from the spleen and VAT were treated with phorbol myristate acetate (PMA, 50 ng/mL) and ionomycin (500 ng/mL) (Sigma-Aldrich) for 4.5 h. Then the cytokines in specific cell populations were analyzed. The cells were stained at 4 °C for 30 min or 1 h for surface and intracellular staining, respectively, followed by detection using the FACS Canto II (BD Biosciences) system and analysis of the data using FlowJo (Tree Star, version 10.0).

#### Real-time quantitative PCR

RNA of adipose tissue was extracted using TRIzol reagent (Life Technologies, Grand Island, NY). 50 mg adipose tissue/sample was ground into homogenization in liquid nitrogen before RNA extraction. We employed the QuantiTect Reverse Transcription kit (Qiagen, Hilden, Germany) to reverse mRNA into cDNA. For quantitative RT–PCR (qPCR), aliquots of cDNA were employed for Fast SYBR green qPCR (Applied Biosystems, Foster City, USA) and quantified with the QuantStudio 5 Real-Time PCR System (Applied Biosystems, Foster City, USA). The sequence of primers are summarized in the Supplementary Table 1.

### ELISA

Serum insulin concentrations of Lkb1^f/f^ and CD11c^Cre^Lkb1^f/f^ mice were detected by ELISA (EZRMI-13 K, Merckmillipore) following the manufacturer's instructions. The blood collections are random and without fasting in Fig. 3E. The insulin secretion experiment followed the IPGTT in Fig. 4F.

### Cholesterol, triglyceride, and glucose measurements

Total cholesterol and triglycerides were measured using the Sigma kit, and free cholesterol was measured using the Wako kit (Richmond, VA). Glucometers (Bayer, Elkhart, IN, USA) was used to measure blood glucose levels. The blood collections are random and without fasting in Fig. 3G.

### Isolation and in vitro differentiation assay of mice preadipocytes

Epididymal fat pads from male Lkb1^f/f^ mice were surgically dissected. The AT digestion and extraction methods were the same as those described above for SVF isolation. The suspended mature adipocytes were discarded, and the precipitated interstitial vascular fraction was cultured in DMEM/F-12 supplemented with 10% FBS. After two-day cell confluence (designated as day 0), we washed and discarded the suspended cells, and the confluent cells are mostly the preadipocytes. Preadipocyte differentiation was carried out with DMEM/F-12 containing 10% FBS and three kinds of induction agents (0.75 mM isobutylmethylxanthine (IBMX), 1.5 μM dexamethasone, and 1.5 μg/mL insulin) for three days. The cells were then incubated in a 10% FBS/DMEM/F-12 culture medium with 1.5 μg/mL insulin and 25, 50 ng/mL IFN-γ or IL-17A, and the IFN-γ or IL-17A-free group was used as the control. The culture medium with or without IFN-γ or IL-17A was refreshed every other day. On day 14, the cells were washed twice with phosphate-buffered saline (PBS) and stained with Oil Red O solution for 20 min at room temperature. Stained Oil Red O was eluted with isopropanol after the cells were washed, and the differentiation capacity of preadipocytes was determined by measuring the optical density (OD) at 510 nm [32, 33].

### Detection of mice preadipocyte size, proliferation, and apoptosis

After 24-h adherent preadipocytes enrichment, the culture medium was replaced with a complete medium containing 50 ng/mL IFN-γ or IFN-γ-free medium for 72 h. Then, the cells were digested with trypsin and harvested for downstream flow cytometry analysis. The FITC Annexin V Apoptosis Detection Kit with PI (640,914, Biolegend) was used to detect apoptosis, while proliferation was determined by the expression of Ki-67 (652,409, Biolegend).

### Statistics

Differences between the two groups were determined using an unpaired, two-tailed Student’s *t*-test, and one-way ANOVA was performed to compare more than two groups using Graph Pad Prism 7 software. Values are presented as mean ± SEM or mean ± SD. Statistical significance was set at* P* < 0.05 (^*^P < 0.05, ^**^P < 0.01, ^***^P < 0.001, ^****^P < 0.0001).

## Results

### CD11c^Cre^Lkb1^f/f^ mice exhibit obesity resistance in the diet-induced obesity model.

**Fig. 1:**
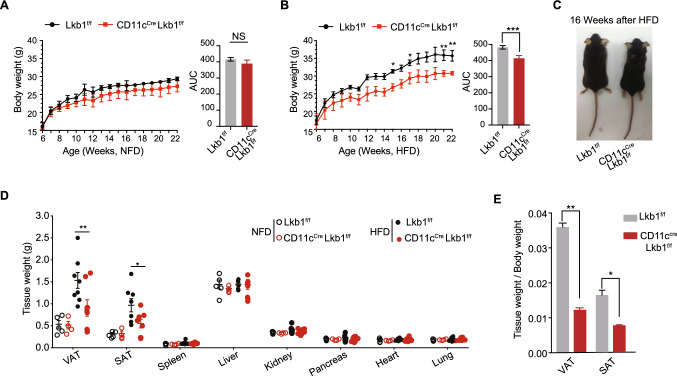
Loss of Lkb1 in CD11c^+^ cells leads to decreased weight gain of adipose tissue in the diet-induced obesity model. **A** Body weight monitoring of mice in the normal fat diet group beginning at week 6 (*n* = 4). And statistical analysis of the area under the curve (AUC). **B** Body weight of mice in the high-fat diet group beginning at week 6 (*n* = 4). And statistical analysis of AUC. **C** Representative images of Lkb1^f/f^ and CD11c^Cre^Lkb1^f/f^ mice after 16 weeks of HFD.** D** Tissue weight of VAT, SAT, spleen, liver, kidney, pancreas, heart, and lung from Lkb1^f/f^ and CD11c^Cre^Lkb1^f/f^ mice after 16 weeks NFD or HFD feeding. **E** Weight of visceral adipose tissue (VAT) and subcutaneous adipose tissue (SAT) standardized by body weight. The results are presented as the mean ± SD, *n* = 4–10, ^*^P < 0.05, ^**^P < 0.01, ^***^P < 0.001, ^****^P < 0.0001. Black asterisks represent multiple *t*-test (**A**, **B**, **D**, **E**) and the red asterisk represent two-way ANOVA (**B**)

**Fig. 2:**
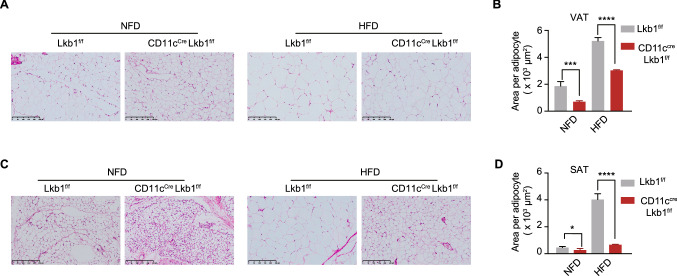
Limited expansion of adipocyte in CD11c^Cre^Lkb1^f/f^ mice is partially responsible for the weight-gain resistance of adipose tissue. **A**, **C** Representative H&E staining images of VAT and SAT from the indicated group. **B**, **D** Statistical analysis of mean size of adipocytes in VAT and SAT from the indicated group. Every counted adipocyte area was determined by at least an unbiased 100 adipocytes per image, and each indicated group selected four images. This result was analyzed by ImageJ. The results are presented as the mean ± SD, ^*^P < 0.05, ^**^P < 0.01, ^***^P < 0.001, ^****^P < 0.0001, by Student's t-test

CD11c^+^ myeloid cells are active members in obese adipose tissue [16, 17, 34]. Lkb1 is considered a dominant energy sensor and mediator of cellular response by activation of AMP-activated protein kinase (AMPK) [35]. We therefore compared the expression of *LKB1* and *AMPKA* in ATDC and ATM among fasting *vs* fed and NFD *vs* HFD (Fig. S1A-B). Among the fasting *vs* fed cohort, the results showed that these two genes in ATDC rather than ATM sensitively responded to starvation, in which *LKB1* increased significantly while the *AMPKA* on the opposite. As for the NFD *vs* HFD cohort, we observed a significant increase in *AMPKA*’s expression in ATDC rather ATM. We further compared the expression of *LKB1* between splenic DC and ATDC, the splenic DC has a higher level of LKB1 than ATDC, meanwhile, both splenic DC and ATDC has a higher level of LKB1 than their macrophage counterparts (Fig. S1C). To investigate the role of Lkb1 in adipose tissue CD11c^+^ cells, we thus generated *Lkb1* conditional knockout CD11c^Cre^Lkb1^f/f^ mice. The knockout deficiency of Lkb1 in CD11c^+^ cells was determined in our previous study at protein levels. We further tested the *Lkb1* in CD11C^+^ CD64^-^ splenic DC and CD11C^+^ CD64^+^ splenic macrophages from the CD11c^Cre^Lkb1^f/f^ and Lkb1^f/f^ mice at mRNA levels, which demonstrate that the knockout efficiency is significant (Fig. S1D). We then established a high fat diet-induced obesity (DIO) model using CD11c^Cre^Lkb1^f/f^ and Lkb1^f/f^ mice. Notably, CD11c^Cre^ Lkb1^f/f^ mice gained lower body weight than their wild-type Lkb1^f/f^ littermates, especially when exposed to HFD (Fig. 1A–C). With interest, we weighed various tissues and observed that adipose tissues (VAT and SAT) but not liver are responsible for the lower body weight of CD11c^Cre^Lkb1^f/f^ than Lkb1^f/f^ mice upon HFD challenge (Fig. 1D, E). The histology examination further indicated that the average area of adipocytes from VAT and SAT in CD11c^Cre^Lkb1^f/f^ mice was significantly smaller than that in Lkb1^f/f^ mice when fed with HFD (Fig. 2A–D). In addition, the livers from both genotypic mice have generated obvious steatosis and there is no significant difference in liver steatosis between CD11c^Cre^Lkb1^f/f^ and Lkb1^f/f^ mice (Fig. S1H). And we calculated the liver versus body weight, there is no difference observed between NFD and HFD (Fig. S1I). These results collectively suggested that CD11c^Cre^ Lkb1^f/f^ mice displayed a fat gain resistant phenotype in the DIO model featured with limited expansion of adipocytes.

### CD11c^Cre^Lkb1^f/f^ mice display normal energy intake and expenditure but a complex expression of *UCP1*.

To determine whether the body weight difference between CD11c^Cre^Lkb1^f/f^ and Lkb1^f/f^ mice is derived from the difference energy intake or energy expenditure [36], we measured the food intake and rectal temperature. The results showed that there is no difference between two genotypic mice under HFD feeding in both perspectives (Fig. S1E-F). Additionally, we examined the expression of uncoupling protein 1 (*UCP1*) in VAT from HFD fed mice and BAT from NFD fed mice. Consistent with the previous knowledge [37], *UCP1* is specifically expressed in BAT as the expression levels in BAT was 100 times higher than VAT. Additionally, we observed that BAT from CD11c^Cre^Lkb1^f/f^ mice had a significant and 1.44 times higher than Lkb1^f/f^ mice (Fig. S1G). However, CD11C^cre^ LKB1^f/f^ mice had significantly lower expression of *UCP1* in VAT (Fig. S1G, I) compared to Lkb1^f/f^ mice*.*

### CD11c^Cre^Lkb1^f/f^ mice display impaired glucose metabolism on HFD feeding.

**Fig. 3:**
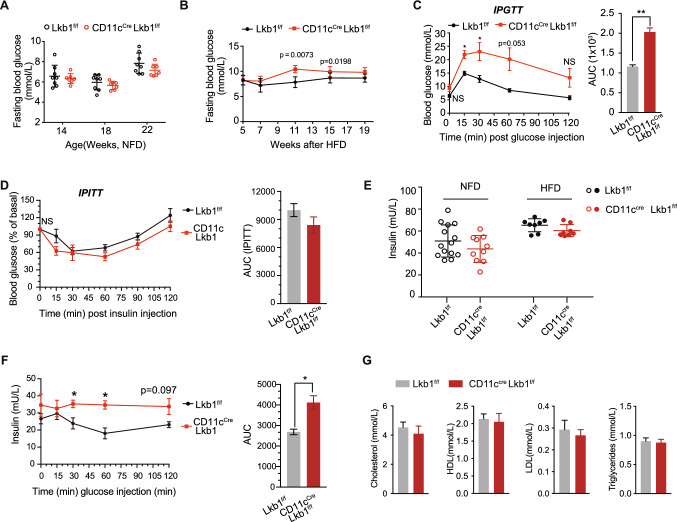
Lkb1 deficiency in CD11c^+^ myeloid cells impairs glucose tolerance and improves insulin sensitivity while does not alter systemic lipid metabolism. **A**, **B** Fasting blood glucose of Lkb1^f/f^ and CD11c^Cre^Lkb1^f/f^ mice on NFD (**A**) and HFD (**B**) at indicated time point. **C** Intraperitoneal glucose tolerance test (IPGTT) of mice after 16 weeks of HFD feeding. And statistical analysis of the area under the curve (AUC) of the IPGTT from Lkb1^f/f^ and CD11c^Cre^Lkb1^f/f^ mice. **D** Intraperitoneal insulin tolerance test (IPITT) of mice after 20 weeks of HFD feeding and statistical analysis of AUC. **E** Insulin detection of mice fed with NFD or HFD. **F** Insulin detection during IPGTT experiment in Supplementary Fig. 2E and statistical analysis of AUC. **G** Cholesterol, high-density lipoprotein (HDL) low-density lipoprotein (LDL), and triglyceride were detected in mice fed with HFD. The results are presented as the mean ± SD (**A**, **B**) and the mean ± SEM. **C–F**, *n* = 4–10, ^*^P < 0.05, ^**^P < 0.01, by Student's *t*-test

Obesity and systemic metabolism disorder are closely associated [8, 38]. To explore whether the obesity resistance of CD11c^Cre^Lkb1^f/f^ mice has protective roles in systemic metabolism, we performed a series of tests regarding glucose and lipid metabolism. There is no significant difference in fasting blood glucose between NFD-fed CD11c^Cre^Lkb1^f/f^ and Lkb1^f/f^ mice, whereas CD11c^Cre^Lkb1^f/f^ mice had a moderately higher levels of fasting blood glucose than Lkb1^f/f^ mice post 11 and 15 weeks of HFD feeding. However, the difference was narrowing down after 11 weeks post HFD feeding (Fig. 3A, B). We further performed intraperitoneal glucose tolerance testing (IPGTT), which showed that HFD induces a higher level of blood glucose and area under the curve (AUC) in CD11c^Cre^Lkb1^f/f^ mice than that in Lkb1^f/f^ mice (Fig. 3C), meanwhile, CD11c^Cre^Lkb1^f/f^ mice had a tendency to higher glucose intolerance but without a significant difference compared with Lkb1^f/f^ mice (Figure S2A). On the other hand, there is no significant difference in the intraperitoneal insulin tolerance test (IPITT) between CD11c^Cre^Lkb1^f/f^ and Lkb1^f/f^ mice in both NFD and HFD conditions (Fig. 3D, S2B). Additionally, CD11c^Cre^Lkb1^f/f^ mice had slightly lower levels of serum insulin than Lkb1^f/f^ mice both in NFD and HFD conditions with no statistical significance (Fig. 3E). Considering the body weight itself might affect the body metabolism, we therefore measured the IPGTT and ATDC composition in an age and weight-matched cohort. The results showed that CD11c^Cre^Lkb1^f/f^ mice still have significant glucose intolerance compared to the Lkb1^f/f^ mice (Fig S2 C-E), but the differences narrow down when compare with the random cohort (Fig. 3D). Meanwhile, we measured the insulin levels during the IPGTT to determine the function of islet in weight matched Lkb1^f/f^ and CD11c^Cre^Lkb1^f/f^ mice fed with HFD (Fig. 3F, S2C-E). The results showed that KO mice secreted a higher level of insulin but impaired to reduce glucose compared to WT mice (Fig. 3F, S2E). As for the lipid indices, no significant differences between CD11c^Cre^Lkb1^f/f^ mice and Lkb1^f/f^ mice, including cholesterol, high-density lipoprotein, low-density lipoprotein, and triglyceride (Fig. 3F). Taken together, these results indicated that obesity-resistant CD11c^Cre^Lkb1^f/f^ mice could normalize fat-induced insulin resistance to some extent, slightly increases glucose intolerance, and has no apparent effects on systemic lipid metabolism in diet induced obesity model.

### Loss of Lkb1 in CD11c^+^ cells specifically restrains the accumulation and CD80’s expression of ATDC in the DIO model

**Fig. 4:**
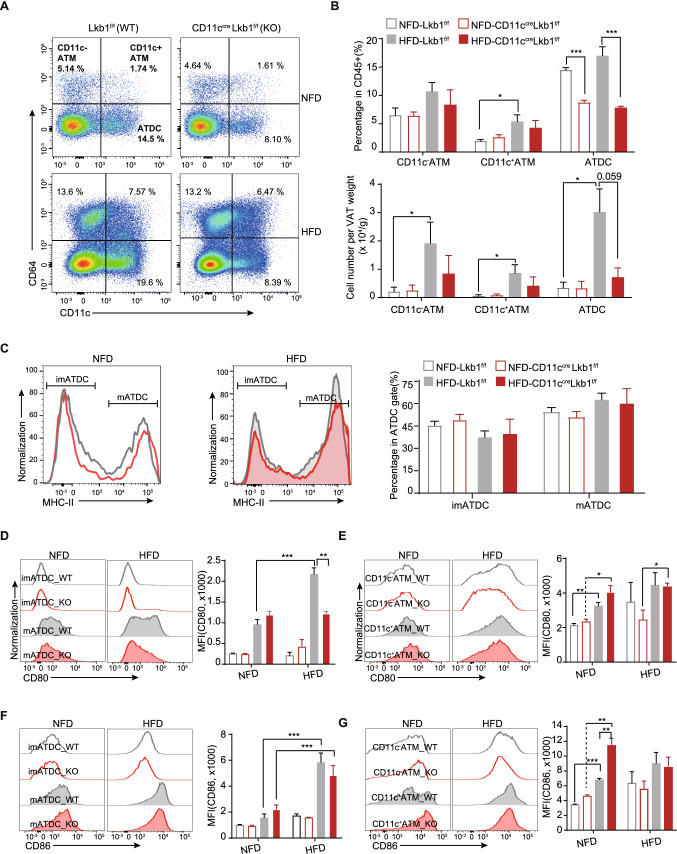
Lkb1 deficiency in CD11c^+^ myeloid cells limited the accumulation and expression of CD80 on ATDC. **A**, **B** Representative flow plot and statistical analysis of adipose tissue CD45^+^ cells from Lkb1^f/f^ (WT) and CD11c^Cre^Lkb1^f/f^ (KO) mice after 16 weeks’ NFD or HFD feeding. **C** Representative overlay histograms of the ATDC and statistical analysis of the composition of ATDC subsets from WT and KO mice after 16 weeks’ NFD or HFD feeding. **D**, **E** Representative overlay histograms of the ATDC and ATM subsets and statistical analysis of the MFI of CD80 in indicated groups. **F**, **G** Representative overlay histograms of the ATDC and ATM subsets and statistical analysis of the MFI of CD86 in indicated groups. imATDC, immature ATDC; mATDC, mature ATDC. The results are presented as the mean ± SEM, ^*^P < 0.05, ^**^P < 0.01, ^***^P < 0.001, ^****^P < 0.0001, by Student's *t*-test. Data are representative of at least three independent experiments

Deletion of the *Lkb1* gene in CD11c^+^ cells is the primary variable between CD11c^Cre^Lkb1^f/f^ and Lkb1^f/f^ mice. Therefore, we followed the reported gating strategy combining CD11c and CD64 to determine the effects of Lkb1 on the adipose tissue CD11c^+^ dendritic cells and macrophages (Fig. S5A) [14]. The results showed that ATDC is the major component among the CD11c^+^ myeloid cells in Lkb1^f/f^ mice fed with NFD or HFD. In addition, HFD-fed Lkb1^f/f^ mice had a higher percentage and normalized cell number of CD11c^−^ATM, CD11c^+^ recruited ATM, and ATDC than NFD-fed Lkb1^f/f^ mice (Fig. 4A, B), which is consistent with the observations in previous DIO studies [10, 14, 34, 39]. Whereas the percentage and number of ATDC rather than CD11c^+^CD64^+^ATM in CD11c^Cre^Lkb1^f/f^ mice were significantly lower than their Lkb1^f/f^ counterparts (Fig. 4A, B). The similar results was also observed in an age and weight-matched cohort with a slightly lower difference (Figure S2F). However, SAT from CD11c^Cre^Lkb1^f/f^ mice has a lower percentage of ATM and ATDC than that from Lkb1^f/f^ mice and has no difference under HFD exposure (Fig. S3A, B). MHC-II is recognized as the mature marker of dendritic cells [40, 41], we thus detected the expression of MHC-II in ATDC. And we observed the percentage of MHC-II^+^ mature ATDC (mATDC) significantly increased after HFD feeding but there were no differences between these two kinds of genotypic mice in both diet groups (Fig. 4C). Furthermore, we examined the expression of co-stimulatory molecules, CD80 and CD86, on ATDC and ATM (Fig. 4D–G). The results demonstrated that immature ATDC (imATDC) sparely expressed CD80 and CD86 whereas mature ATDC (mATDC) were highly expressed and elevated on HFD exposure. Intriguingly, the upregulation of CD80 in mATDC from CD11c^Cre^Lkb1^f/f^ mice was significantly impaired. ATM had higher levels of these two molecules while there is no impaired expression of CD80/86 in ATM from CD11c^Cre^Lkb1^f/f^ mice. To validate these observations in ATDC and explore whether these differences are tissue-specific, we utilized another gating strategy (Figs. S4A-C, S5B) and tested splenic DC (Fig. S4D-E). The results further elaborated the above observations in ATDCs and showed that there was no significant difference between splenic DC in terms of cell number and the expression of CD80/CD86. Since the previous reports demonstrated the alteration of IL1A, CCL5, and OX40 in the Lkb1-deficient DC and expression of those genes are also well known to regulate adipose tissue inflammation in obesity [42–45], we examined the expression of those essential genes on ATM and ATDC. The results showed that ATDC from CD11c^Cre^Lkb1^f/f^ has lower levels of *CCL5* and *OX40* while higher levels of *IL1A* compared to Lkb1^f/f^ ATDC. The difference in ATM from two genotypic mice is relatively minor in which only *OX40* displays a lower expression from CD11c^Cre^Lkb1^f/f^ mice (Fig. S1K-M). While the overall levels of *IL6* in VAT is comparable between WT and KO mice (Fig. S1J). Therefore, we concluded that HFD feeding favorably induces adipose tissue-specific changes in CD11c^+^ ATDC, and loss of Lkb1 in these cells curtails the accumulation and the expression of CD80, *CCL5*, *OX40* in ATDC.

### Lkb1 deficiency in CD11c^+^ cells specifically reshapes adipose tissue T cell profile upon HFD exposure.

**Fig. 5:**
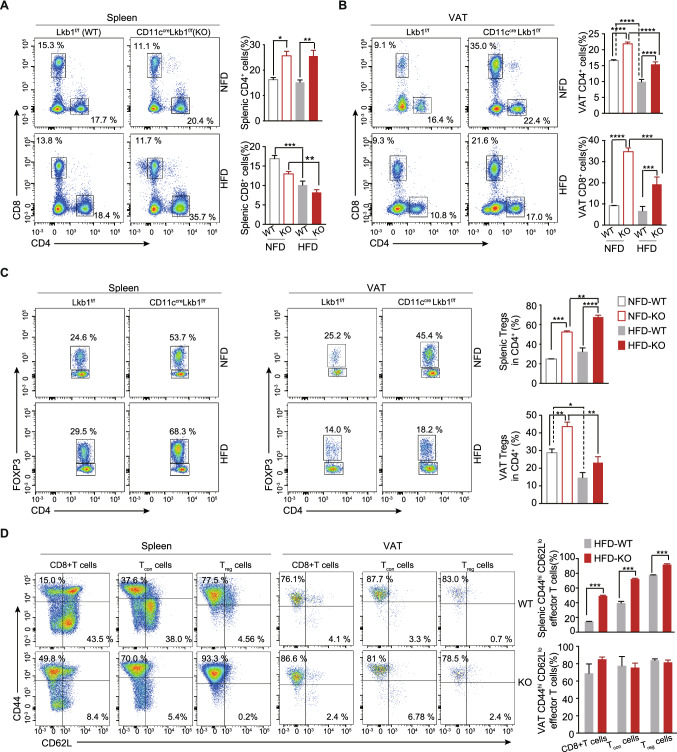
Loss of Lkb1 in CD11c^+^ myeloid cells leads to the shape of a T cell-abundant immune profile in the VAT. **A**, **B** Representative flow plot and statistical analysis of splenic (**A**) and VAT (**B**) T cells from Lkb1^f/f^ (WT) and CD11c^Cre^Lkb1^f/f^ (KO) mice with NFD and HFD. **C** Representative flow plot and statistical analysis of splenic and VAT T_regs_ from Lkb1^f/f^ and CD11c^Cre^Lkb1^f/f^ mice with NFD and HFD. **D** Representative flow plot and statistical analysis illustrating effector/memory phenotype of splenic and VAT CD8^+^, T_con_, and T_regs_ from the two genotypes of mice fed with HFD. The results are presented as the mean ± SD, ^*^P < 0.05, ^**^P < 0.01, ^***^P < 0.001, ^****^P < 0.0001, by Student's t-test. Data are representative of at least three independent experiments

To investigate whether loss of Lkb1 in CD11c^+^ cells remodels the T cell landscape in AT and its tissue specificity, we examined T cells from the spleen and VAT. As for the spleen, HFD significantly reduced the percentage of CD8^+ ^T cells but not CD4^+ ^T cells. In addition, the percentage of CD4^+ ^T cells from CD11c^Cre^Lkb1^f/f^ mice was significantly higher than those from Lkb1^f/f^ mice independent of the type of diet, whereas CD8^+^ T cells had a lower percentage with no significance (Fig. 5A). Different from the spleen, both VAT CD4^+^ and CD8^+^ T cells from CD11c^Cre^Lkb1^f/f^ mice were significantly higher than that from Lkb1^f/f^ mice (Fig. 5B, S2G). As for the T_reg_ cell, consistent with our previous study [26], the proportion of splenic and VAT T_regs_ from CD11c^Cre^Lkb1^f/f^ mice was significantly higher than that from Lkb1^f/f^ mice in the NFD group. Unexpectedly, this VAT T_regs_ advantage in CD11c^Cre^Lkb1^f/f^ mice narrowed down on HFD exposure (Fig. 5C, S2G). On the contrary, the proportion of splenic T_regs_ increased (Fig. 5C). We further detected the expression of the Helios, a good marker of mice thymic T_regs_ [46], in VAT CD4^+^ FOXP3^+^ T cells as you mentioned. The results showed that there is no difference between CD11c^Cre^Lkb1^f/f^ and Lkb1^f/f^ mice (Fig. S2I). As for the T cell activation status, we observed that splenic T cells from CD11c^Cre^Lkb1^f/f^ mice were markedly activated (CD44^hi^CD62L^low^) compared to those from Lkb1^f/f^ mice with HFD feeding. Meanwhile, VAT had a high level (> 75%) of CD44^hi^CD62L^low^ effector T cells, and they were comparable between the two genotypic mice fed with HFD (Fig. 5D, S2H). To determine whether Lkb1 knockout ATDC has depot-specific effects regarding the T cell composition, we further detected the T cell composition in SAT. The results showed that CD8 T cells and T_reg_ cells had a higher percentage in KO mice under NFD and HFD condition, respectively (Fig. S3C-D). Overall, these results indicated that Lkb1 deletion in CD11c^+^ cells shaped a higher level of the activation status of splenic T cells and T cell-abundant VAT immune microenvironment, and HFD narrowed down the VAT T_regs_ advantages in CD11c^Cre^Lkb1^f/f^ mice.

### Lkb1 deficiency in CD11c^+^ cells tips the IL-17A and IFN-γ balance towards IFN-γ in various immune cells upon HFD challenge

**Fig. 6:**
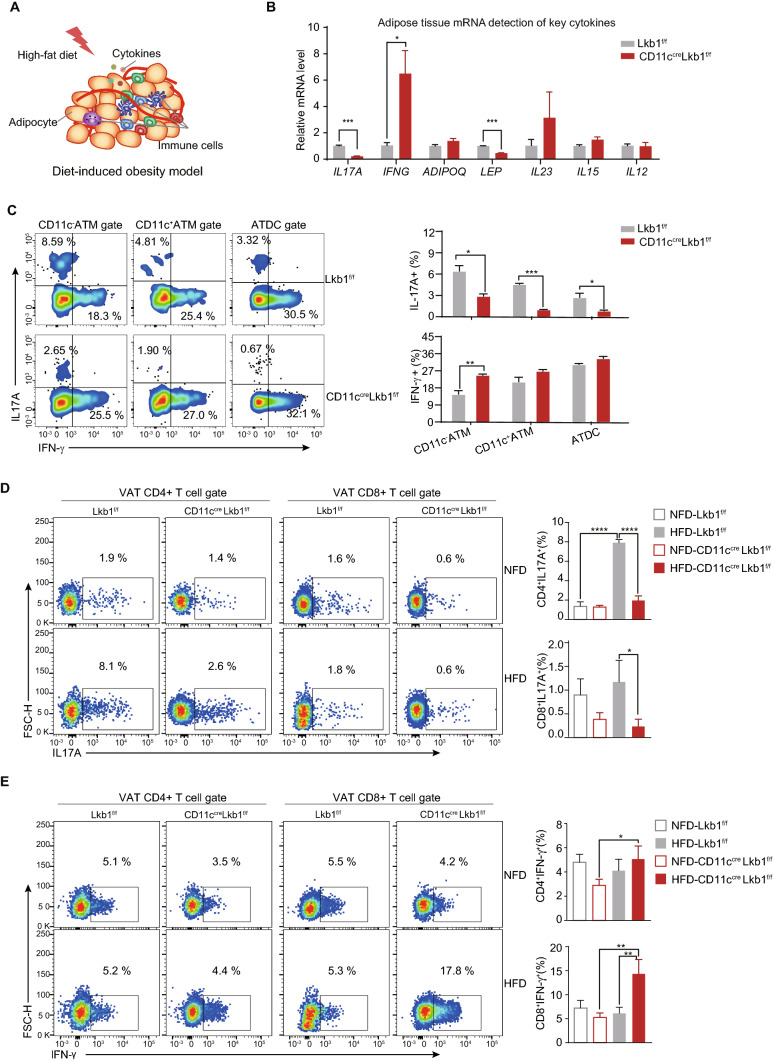
Deficiency of Lkb1 tips IL-17A/IFN-γ towards IFN-γ in both adipose-tissue T cells and ATMs. **A** Schematic represents major components of the adipose tissue in DIO model. **B** Real-time qPCR analysis of the indicated cytokine expression in adipose tissue from Lkb1^f/f^ and CD11c^Cre^Lkb1^f/f^ mice fed with HFD. **C** Representative flow plot (left) and statistical analysis (right) of the expression of IL-17A and IFN-γ in CD45^+^ ATM and ATDC cells from Lkb1^f/f^ and CD11c^Cre^Lkb1^f/f^ mice fed with HFD. **D** Representative flow plot (left) and statistical analysis (right) of the expression of IL-17A in CD4 and CD8 T cells from Lkb1^f/f^ and CD11c^Cre^Lkb1^f/f^ mice fed with HFD. **E** Representative flow plot (left) and statistical analysis (right) of the expression of IFN-γ in CD4 and CD8 T cells from Lkb1^f/f^ and CD11c^Cre^Lkb1^f/f^ mice fed with HFD. The results are presented as the mean ± SD, ^*^P < 0.05, ^**^P < 0.01, ^***^P < 0.001, ^****^P < 0.0001, by Student's *t*-test. Data are representative of at least three independent experiments

Except for the immune cells, cytokines as the pivotal factors that affect the AT immune microenvironment and the metabolic outcomes of DIO **(**Fig. 6A). Therefore, we performed real-time qPCR of total adipose tissue to detect the mRNA levels of several well-recognized cytokines (Fig. 6B, S1J). Compared to Lkb1^f/f^ mice, adipose tissue from CD11c^Cre^ Lkb1^f/f^ expressed higher levels of *IFNG*, whereas *IL-17A* and *LEP, TNFA* were significantly lower (Fig. 6B, S1J). Leptin is well defined and mainly derived from adipocytes. Given these indications, we further conducted intracellular cytokine detection of VAT resident immune cells to testify the IFN-γ and IL-17A imbalance at the protein level and figure out their cellular origins (Fig. 6C–E). Consistent with the mRNA observations, we found that IFN-γ and IL-17A imbalance existed in various immune cells. More specifically, myeloid cells including CD11c^−^ ATM, CD11c^+^ ATM, and ATDC from CD11c^Cre^Lkb1^f/f^ mice had significantly lower IL-17A, whereas IFN-γ was higher in these myeloid populations, in which the CD11c^−^ATM was the most significant (Fig. 6C). As for the CD45^+^CD11c^−^CD64^−^ cells, we detected these two cytokines in T cells (Fig. 6D, E). In Lkb1^f/f^ mice, HFD feeding mainly promoted the generation of CD4^+^T derived IL-17A (Th17 cells) rather than IFN-γ. Surprisingly, CD11c^Cre^ Lkb1^f/f^ mice on the opposite, HFD feeding rarely upregulated the generation of Th17 but significantly induced higher levels of IFN-γ. Thus, compared with Lkb1^f/f^ mice, a lower percentage of IL-17A^+^ and a higher percentage of IFN-γ^+^ CD4/CD8 T cells from CD11c^Cre^ Lkb1^f/f^ mice were detected. The IL-17A and IFN-γ are mainly from CD4 and CD8 T cells, respectively, the corresponding significant differences as well. Taken together, these results collectively suggest that Lkb1 deficiency in CD11c^+^ cells tips the IL-17A and IFN-γ balance towards IFN-γ in VAT T cells and macrophages upon HFD challenge.

### IFN-γ ex vivo promotes apoptosis of preadipocytes and inhibits their adipogenesis while IL-17A promotes their adipogenesis

**Fig. 7:**
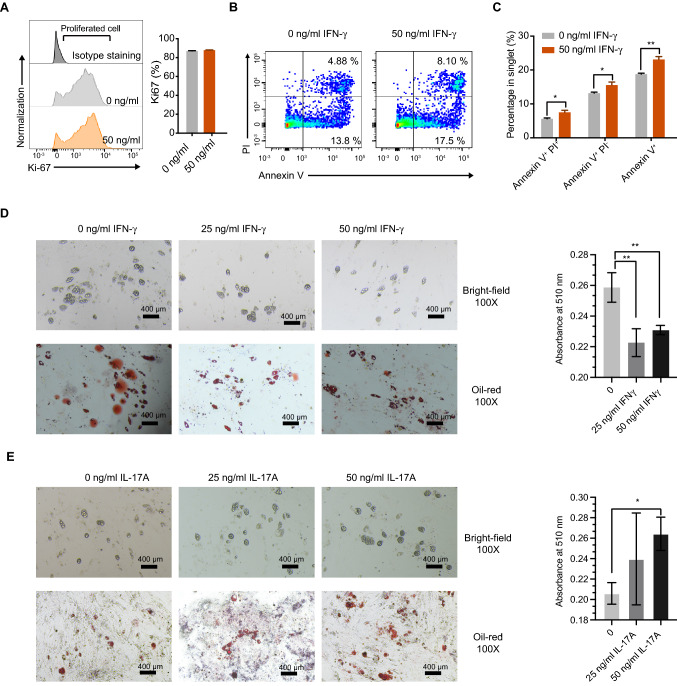
In vitro IFN-γ promoted apoptosis and inhibited differentiation of mice preadipocytes but had no significant effect on proliferation and cell size. **A** Representative overlay histogram (left) and statistical analysis (right) of proliferation index Ki-67 in preadipocytes from Lkb1^f/f^ mice in vitro cultured 72 h with 0 and 50 ng/ml IFN-γ. **B** Representative flow plot illustrating the apoptosis analysis of preadipocytes from Lkb1^f/f^ mice treated with 0 and 50 ng/ml IFN-γ. **C** Statistical analysis of early apoptosis, late apoptosis, and total apoptosis of preadipocytes from Lkb1^f/f^ mice treated with 0 and 50 ng/ mL IFN-γ. **D**, **E** Representative images and statistical analysis of cell morphology (up) and Oil red “O” staining (bottom) at the end of *in-vitro* differentiation (Day 14) with indicated conditions of the preadipocytes from Lkb1^f/f^ mice. The results are presented as the mean ± SEM, ^*^P < 0.05, ^**^P < 0.01, ^***^P < 0.001, ^****^P < 0.0001, by One-way ANOVA. Data are representative of at least three independent experiments

To further testify the role of IFN-γ and IL17A in AT, we performed in vitro functional assay of IFN-γ and IL17A on preadipocytes. The results showed no difference in the Ki-67^+^ cell rate and cellular size between the 50-ng/mL IFN-γ group and the IFN-γ-free group (Fig. S2J, 7A). Compared to the IFN-γ-free control group, the addition of 50-ng/mL IFN-γ in the preadipocytes culture system after 72 h significantly increased the percentage of Annexin V^+^ apoptosis cells, including the early Annexin V^+^ PI^−^ and late Annexin V^+^ PI^+^ apoptosis cells (Fig. 7B, C). The in vitro differentiation assay of mice preadipocytes showed that the lipid formation was significantly decreased in both 25- and 50-ng/mL IFN-γ group compared to that in the IFN-γ-free group and displayed a dose dependent effect (Fig. 7D). On the contrary, there are more adipocytes differentiated in IL-17A treated group (Fig. 7E). In summary, the ex vivo experiments indicate that IFN-γ promoted apoptosis and inhibited the differentiation of preadipocytes, with no significant effects on their proliferation and cellular size. Meanwhile, IL-17A has a positive effect on the adipogenesis.

## Discussion

Mounting evidences have elaborated adipose immune status is closely associated with obesity and threatening metabolism disorders [1, 8, 38]. CD11c^+^ myeloid cells are involved in overnutrition associated immune-metabolic disorders [14, 16, 17, 39]; therefore, understanding of the underlying molecular basis becomes essential in developing intervention strategies for these threatens. Previous studies have revealed the critical role of the nutrient sensor Lkb1 in controlling the function of splenic and lymphoid CD11c^+^ dendritic cells [26–28, 47]. However, the specific role of Lkb1 in fat CD11c^+^ cells, especially under the high-fat diet exposure, has not been addressed. The present study has elucidated how Lkb1 in fat CD11c^+^ cells modulate the adipose immune environment and affect the outcomes of diet-induced obesity (DIO).

DIO is the most widely investigated model of type 2 diabetes with hyperglycemia, glucose intolerance, and insulin resistance [8, 10, 16, 39]. There is no significant metabolic dysfunction in CD11c^Cre^ Lkb1^f/f^ mice on NFD feeding compared to Lkb1^f/f^ mice. Upon HFD exposure, different from normal mice CD11c^Cre^ Lkb1^f/f^ mice had a lower adipose-weight gain and smaller expansion of adipocytes accompanying significantly impaired glucose tolerance. Moreover, according to the metabolic analysis from body weight matched cohort, CD11c^Cre^ Lkb1^f/f^ mice still have significant glucose intolerance compared to the Lkb1^f/f^ mice, but the differences narrow down slightly when compared with the random cohort, indicating the body composition partially participates in the metabolic, and also suggesting that there are other potential mechanisms in the downstream of Lkb1 deficiency in CD11c^+^ cells involved in the regulation of metabolic status in the CD11c^Cre^ Lkb1^f/f^ mice.

We observed no significant changes in obesity resistant CD11c^Cre^ Lkb1^f/f^ mice regarding the calories intake and rectal temperature. At gene expression level, *UCP1*, mainly expressed on brown adipose tissue (BAT) and brown-like beige cells [37], upregulates thermogenesis and energy expenditure and therefore protect mice from obesity [48, 49]. The BAT CD11c^Cre^ Lkb1^f/f^ mice has 1.44 times higher expression of *UCP1* than Lkb1^f/f^ mice under the NFD feeding, which might be associated with the fat gain resistance phenotype of CD11c^Cre^ Lkb1^f/f^ mice. Meanwhile, the morphology, expression of *UCP1* and oxygen consumption in isolated BAT from DIO model mice are needed to address. Adipose tissue browning is a reversible process that the brown-like beige cells recruit from progenitor cells and transdifferentiate from white adipocytes, which is critical for thermogenic demand and metabolic healthy [50]. The extremely lower expression of *UCP1* in CD11c^Cre^ Lkb1^f/f^ VAT might indicate that there is a smaller number of beige cells within VAT or lower levels of adipose tissue browning in CD11c^Cre^ Lkb1^f/f^ mice. And whether these differences in *UCP1* expression ascribe to the obesity resistance and impaired glucose tolerance is warranted to future studies.

Adipose tissue and liver have high risks in steatosis upon HFD exposure [51, 52]. The gained weight of liver varies and the liver/body weight ratio always decreases in different studies with different diet regimens [51–53]. Similar with a previous study [53], we did not observe significant liver weight gain and higher liver/body weight ratio in our DIO model. Only the adipose tissue has significantly increased weight among multiple tissues, suggesting the body weight gain of obese mice are mainly from adipose tissue. The increase in hypertrophy (the size) and hyperplasia (the number) of adipocyte causes fat-weight gain and storage the excess calories under HFD exposure [54]. Additionally, it is reported that hypertrophy is strongly correlated with diet and occurs earlier, whereas hyperplasia is more dependent on genetics [55]. Both the CD11c^Cre^ Lkb1^f/f^ and Lkb1^f/f^ mice are obesity-prone C57BL/6 (C57) mouse strain, therefore we could exclude the genetic background on the hyperplasia between them. Hypertrophy of adipocyte is basically determined by energy intake and expenditure, which is regulated by adipokines and immune cytokines. The most well-known adipokines are leptin and adiponectin. Obese people and mice has high levels of leptin that inhibits food intake while adiponectin is responsible for energy metabolism [56, 57]. The fat gain resistant CD11c^Cre^ Lkb1^f/f^ mice had remarkably lower levels of *LEP* and comparable levels of *ADIPOQ* in adipose tissue than Lkb1^f/f^ mice, indicating leptin and adiponectin are not the direct contributors for the body weight decrease in CD11c^Cre^ Lkb1^f/f^ mice. Previous studies demonstrated that leptin is required for IL-17A production [58, 59], which might indicate the significant lower secretion of IL-17A in adipose tissue possibly due to the lower expression of *LEP* from CD11c^Cre^ Lkb1^f/f^ mice. Notably, a recent study shows that the inhibition of IL-17A axis through inhibition of RORγt-mediated IL-17A production and selective deletion of *IL17RA* in adipocytes could dramatically lead to the reduction in adipocyte size, increased white adipose browning and thermogenesis, as well as promote the expression of diabetogenic and obese genes in adipocytes [60]. Besides, IL-17A could accelerate the inflammatory progression in obese mice via the TBK1/IKBKE pathway using IL-17A deficient mice [61]. Moreover, it is also reported that IL-17A directly promotes the cellular uptake of fatty acids [62]. In agreement with these evidences, our study also observed that the most significantly elevated cytokine in Lkb1^f/f^ mice is IL-17A upon HFD exposure, supporting IL-17A is a strong factor for the pathogenesis of DIO. Compared with IL-17A, IFN-γ secretion in T cells is less sensitive in HFD-fed Lkb1^f/f^ mice. However, the *IL23*-*IFNG* axis are highly activated in fat tissue from HFD-fed CD11c^Cre^ Lkb1^f/f^ mice. And we detected remarkably higher IFN-γ secretion in CD8^+^ T cells and CD11c^−^ ATM from CD11c^Cre^Lkb1^f/f^ mice on HFD challenge. Further tests suggested IFN-γ could promote apoptosis of preadipocytes and inhibit their adipogenesis in vitro, which indicate IFN-γ might inhibit hyperplasia of adipose tissue in vivo. Taken together, the downregulated IL-17A and increased IFN-γ are potentially responsible for the fat gain resistance and smaller adipocytes of CD11c^Cre^ Lkb1^f/f^ mice. However, the functional capacity and gene expression including metabolism, survival, and differentiation of preadipocyte and adipocyte in vivo, and other potential contributors in fat gain resistance of CD11c^Cre^ Lkb1^f/f^ mice await future studies.

Obesity and hypertrophic adipocytes are associated with dysmetabolism, on the contrary, reduced adipocyte size is usually coupled with body weight loss and improved glucose and lipid metabolism [54]. In the present fat gain resistant CD11c^Cre^ Lkb1^f/f^ mice, however, we observed normalized insulin sensitivity after 20 weeks HFD feeding but impaired glucose tolerance after 16 weeks HFD feeding. The initial levels of glucose in IPGTT and IPITT was slightly different which might ascribe to the different time point after HFD feeding and minor difference of blood glucose between two genotypic mice. The impaired glucose tolerance might partially due to the decreased insulin levels. Meanwhile, it might suggest that there are complicating factors involved.

Both IL-17A and IFN-γ are considered proinflammatory contributors in the pathogenesis of metabolic disorders [30, 63, 64]. The dysregulation of IL-17A and IFN-γ in adipose tissue might be one of the critical contributors. Moreover, loss of Lkb1 in CD11c^+^ cells remarkably increases splenic T cell responses under HFD exposure, which is consistent with other reported evidence on CD11c^Cre^ Lkb1^f/f^ mice [26–28, 47]. It is reported that activation of autoreactive T cells is one of critical contributors in autoimmune destruction of beta cells [65]. Therefore, additional experiments to explore whether the overactivated peripheral T cells we observed in CD11c^Cre^ Lkb1^f/f^ mice affect the function of islet including the insulin secretion are needed. In spite of the pathogenic factors, VAT T_regs_ play an essentially positive role in adipose homeostasis [18–21, 23]. In the present study, VAT T_regs_ in NFD-fed CD11c^Cre^Lkb1^f/f^ mice were more abundant than those in Lkb1^f/f^ mice, which indicates Lkb1-deficient CD11c^+^ cells promote the expansion of VAT T_regs_, which is consistent with previous studies [26, 28]. However, this advantage of VAT T_regs_ sharply decreased upon HFD exposure. Despite that, the overall levels of VAT T_regs_ in CD11c^Cre^Lkb1^f/f^ mice was higher than Lkb1^f/f^ mice during the DIO process. In this respect, VAT T_regs_ might play a positive role in metabolic outcomes at some extent. As for the origin of these increased VAT T_regs_, we observed no difference between CD11c^Cre^Lkb1^f/f^ and Lkb1^f/f^ mice, indicating the increased percentage and number of T_regs_ in VAT is more likely from thymic derived rather than tissue induced T_reg_ cells. According to the splenic and VAT T_regs_ examination from both NFD and HFD Fig. 5C, the changes of Treg cells in VAT is less dependent on the splenic T_regs_, suggesting the VAT T_regs_ would be likely tissue-specific, otherwise, LKB1 could regulate ATDC’s chemotaxis effects on T_regs_ during HFD exposure because there is more splenic T_regs_ when VAT T_regs_ declined. Our previous work demonstrated that the expanded T_reg_ pool is mediated by the DC-T_reg_ interaction directly [26], which favors the prior assumption. But we do not know exactly whether these increased T_reg_ cells are from VAT T_regs_ proliferation or migrated from lymphoid tissue or both. Further studies would be needed. Collectively, multiple factors including the imbalance of IL-17A/IFN-γ and decreased expansion of VAT T_regs_ potentially play a role in the unconventional metabolism outcomes of CD11c^Cre^ Lkb1^f/f^ mice.

The factors discussed above are secondary ones, the initial factor is CD11c^+^ cells because the *Lkb1* gene is selectively deleted in CD11c^+^ cells in CD11c^Cre^ Lkb1^f/f^ mice. In addition to adipose tissue dendritic cells (ATDC), CD11c is also expressed on recruited macrophages (ATM). CD64 has been identified as an ideal marker to distinguish ATM from ATDC rather than F4/80 or CD11b and the author demonstrated that ATDCs are independent contributors to adipose tissue inflammation in DIO [14]. In agreement with this study, we found that ATDCs is the dominant CD11c^+^ cell population in adipose tissue by the high-fat diet regimen induced. Moreover, the *Lkb1* expression pattern in ATDC is more sensitive than ATM during fasting. Among the Lkb1^f/f^ mice, ATDC increased in number and the expression of co-stimulation molecules CD80/CD86, and the generation of Th17 was enhanced in HFD fed condition compared to NFD condition, which are consistent with the previous studies [34, 39, 66]. Interestingly, *Lkb1* deficiency more specifically affects ATDC than CD11c^+^ATM regarding the cell number and the expression of CD80, *OX40* and *CCL5*. These evidence might indicate ATDCs are the major contributors to the fat gain resistant phenotype and adipose-tissue immune profile in CD11c^Cre^Lkb1^f/f^ mice. The selective effects of Lkb1 on ATDC are possibly due to the striking difference of the functional profiles between ATDC and ATM. ATM is more capable of storing lipid in the setting of obesity than ATDC [14]. Moreover, it is reported that Lkb1 deficiency results in lipid accumulation and aberrant metabolism/mTOR activation in DCs [28]. The overaccumulation of lipid under HFD condition should be further explored. It might make the Lkb1^−/−^ ATDC more fragile and could be a potential mechanism for the reduction in ATDC. Even though we observed more specific changes in ATDC, we cannot exclude the primary contributions of CD11c^+^ATM on the metabolic and immune phenotypes. Further studies are needed to determine the exact mechanisms underlying Lkb1 on the maintenance of the accumulation and function of ATDCs upon HFD exposure. To determine the role of Lkb1 on ATM, Lysm^cre^Lkb1^f/f^ mice would be needed.

The production of IL-17A is enhanced by ATDCs in both mice and humans, which has clear clinical implications in obesity-associated metabolic disorders [39, 66]. Therefore, the decreased number of ATDCs potentially contribute to the downregulated IL-17A in CD11c^Cre^Lkb1^f/f^ mice. CD80 and CD86 in DCs provided the second co-stimulatory signals necessary for T cells activation and function. The HFD condition and tissue specific CD80 reduction in dendritic cells is interesting. Two previous studies reported that loss of Lkb1 promotes the maturation of splenic DC with higher levels of CD86 but not CD80 [27, 28], which is similar with our observation in spleen, and the CD80’s stagnation of ATDC under HFD exposure. They proposed that deficiency of Lkb1 leads to lipid accumulation, increased glycolysis, enhanced phospholipase C β1, and activation of both mTORC1 and mTORC2 signaling. These cellular changes in metabolism and signaling release DC from quiescence. But why the expression of CD80 in DC did not increase under activation status is not discussed. In the current HFD induced obesity model, the CD80’s stagnation in Lkb1^-/-^ATDC becomes more significant might be partially due to the higher lipid accumulation in ATDC, which suppressed the CD80 regulation pathway. Additionally, AMPK and HIF-1α signaling, the canonical pathways of Lkb1 [67, 68], and other potential signaling are warranted to reveal the detailed mechanisms underlying LKB1 selective CD80 regulation in ATDCs under HFD exposure. The expression of these two Ig B7 superfamily member shares the same regulatory underpinnings such as transcription factor PU.1 [69]. Meanwhile, they also had independent regulatory mechanisms. It is reported that decreasing the expression of CD80 /CD86 tips the Th1/Th2 balance towards the Th1 response featuring higher IFN-γ expression [70]. Whether the impaired CD80 expression contributes to the imbalance of IL-17A/IFN-γ secretion await future studies.

It is well recognized that selective deletion of Lkb1 in CD11c^+^ dendritic cells enhances their capacity to prime effector T cell response and prevailingly expands T_reg_ pools throughout the body [26–28, 47]. Therefore, the decreased number of ATDCs possibly contributed to the decreased advantage of VAT T_regs_ from CD11c^Cre^Lkb1^f/f^ mice under HFD exposure. Additionally, elevated production of the adipokine leptin in obese mice can strongly inhibit the proliferation of T_regs_ by upregulating the cyclin-dependent kinase inhibitor p27 and phosphorylation of ERK1/2 [71]. Our results showed that the *LEP* mRNA of adipose tissue from CD11c^Cre^Lkb1^f/f^ mice was significantly lower than Lkb1^f/f^ mice in DIO. On the other hand, the splenic T_reg_ increased significantly in obese CD11c^Cre^Lkb1^f/f^ mice. Thus, the contributors including ATDC, leptin, and recruitment collectively potentially play a role in the decreased advantage of VAT T_regs_ from fat gain resistant CD11c^Cre^Lkb1^f/f^ mice. Future studies are needed to elucidate the specific mechanisms.

There are several limitations of this study. 1. The overall obesity phenotype including liver weight gain and fasting glucose changed in the DIO model is moderate, Lkb1^f/f^ gene modification or other potential reasons should be identified. 2. We did not validate the direct effects of Lkb1^−/−^ ATDC/ATM on adipose T cells, the co-culture experiments to see antigen presentation and cross-presentation function on T cells by AT CD11c^+^ cells or BMDC from CD11c^Cre^Lkb1^f/f^ and Lkb1^f/f^ mice are needed to be confirmed. 3. The concentration of IFN-γ and IL-17A used in in vitro system is much higher than the in vivo levels, therefore, the in vitro data is far equal to physiological status. Meanwhile, purity of the preadipocytes in the system also should be considered. 4. The knock out deficiency and effects of the CD11c^+^ subsets like CD8^+^ CD11c^+^ cells [72] were not investigated and loss of Lkb1 in these cells could also affect the phenotypes we observed. 5. Adipose tissue fibrosis is a classic pathogenically process in DIO model along with inflammatory responses [73], we did not investigate the influences of loss of Lkb1 in CD11c^+^ cell on this critical process. 6. We observed some phenotypic differences between SAT and VAT but lack the deeper investigations on the Lkb1^−/−^ CD11c^+^ cells on depot-specific effects.

In summary, this study firstly investigated the role of CD11c^+^ cells’ Lkb1 in metabolic disease using conditional knockout CD11c^Cre^Lkb1^f/f^ mice and DIO model. We demonstrate that Lkb1 is an essential metabolic kinase for ATDCs responding to high-fat exposure. Loss of Lkb1 primarily disturbs the accumulation and CD80’s expression of ATDCs, which potentially leads to the dysregulated balance of IL-17A and IFN-γ and reduced VAT T_regs_ advantage of CD11c^Cre^Lkb1^f/f^ mice in DIO model. These immune factors collectively affect the adipocytes and preadipocytes and ultimately determine the overall fat gain resistant outcome of CD11c^Cre^Lkb1^f/f^ mice. Despite adipose tissue specific changes, systemic deletion Lkb1 in CD11c^+^ cells accompanies T cell over activation. In summary, this study reveals the metabolic sensor, Lkb1, is required for CD11c^+^ myeloid cells in response to HFD exposure. Meanwhile, it provides new insights into the role of IL-17A and IFN-γ in diet-induced obesity. These findings suggest specific Lkb1 intervention in ATDC might be a potential strategy for the prevention and treatment of diet induced obesity.

## Supplementary Information

Below is the link to the electronic supplementary material.

Supplementary file1 (XLSX 14 KB)

**Supplemental Fig. 1. (A-B)** mRNA quantitation of *LKB1* **(A)** and *AMPKA* **(B)** of adipose tissue CD11c^+^ cells among fasting, fed, NFD, and HFD conditions. **(C)** The expression of *LKB1* in the adipose tissue and splenic CD11c^+^ subsets. **(D)** The expression of *LKB1* in splenic CD11c^+^ subsets from WT and KO mice. **(E)** Daily monitoring data of accumulated food intake in Lkb1^f/f^ and CD11c^Cre^ Lkb1^f/f^ mice fed with HFD (5.24 kcal/g). **(F)** Monitoring data of rectal temperature in Lkb1^f/f^ and CD11c^Cre^ Lkb1^f/f^ mice in DIO model. **(G)** The *UCP1* expression of VAT and BAT tissue from Lkb1^f/f^ mice fed with NFD or HFD. **(H)** Histological analysis of liver from Lkb1^f/f^ and CD11c^Cre^ Lkb1^f/f^ mice in DIO model. The red arrow represents the infiltrated immune cells; the black arrow represents the hepatic steatosis; the yellow arrow represents ballooning degeneration of hepatocytes. **(I)** Statistical analysis of the liver versus body weight ratio in the indicated groups. **(J)** The expression of *UCP1*, *TNFA*, *IL6* in adipose tissue from WT and KO mice. **(I)** The expression of *IL1A*, *CCL5*, *OX40* in ATM and ATDC from WT and KO mice. The gating strategy of DC and macrophage are based on the expression of CD64 and CD11c as shown in Supplementary Fig. 5. The cells in A-C were sorted from Lkb1^f/f^ mice. WT and KO represent Lkb1^f/f^ and CD11c^Cre^ Lkb1^f/f^ mice, respectively. The results are presented as the mean ± SEM, ^*^P < 0.05, ^**^P < 0.01, ^***^P < 0.001, ^****^P < 0.0001, by Student's t-test. Data are representative of at least three independent experiments. **Supplemental Fig. 2**. **(A)** Intraperitoneal glucose tolerance test (IPGTT) of mice after 16 weeks of NFD feeding. And statistical analysis of the area under the curve (AUC) of the IPGTT from Lkb1^f/f^ and CD11c^Cre^Lkb1^f/f^ mice. **(B)** Intraperitoneal insulin tolerance test (IPITT) of mice after 16 weeks of NFD feeding. **(C-F)** Metabolic and immune analysis of the age and body weight matched cohort from Lkb1^f/f^ and CD11c^Cre^Lkb1^f/f^ mice with 16 weeks of HFD feeding. **(C)** Body weight statistical analysis. **(D)** Fasting glucose. **(E)** IPGTT tests and corresponding statistical analysis of the area under the curve (AUC) of the IPGTT. **(F)** Flow dot plots of ATDC and ATM among CD45^+^ cells. And statistical analysis of the composition of ATDC and ATM. **(G)** Statistical analysis of the absolute number of CD4, CD8, and Treg cells in adipose tissue corresponding to Fig. 5B-C. **(H)** Statistical analysis of absolute number of activated CD4, CD8, and T_reg_ cells in adipose tissue corresponding to Fig.5D. **(I)** Flow dot plot with smooth model represents the expression of Helios in Foxp3^+^ VAT T_regs_ from WT and KO in DIO model. **(J)** Representative overlay histogram (left) and statistical analysis (right) of preadipocytes size in vitro cultured 72 hours with 0 and 50 ng/ml IFN-γ. The comparison of preadipocytes’ size was based on the FSC-A value. The results are presented as the mean ± SEM, ^*^P < 0.05, ^**^P < 0.01, ^***^P < 0.001, ^****^P < 0.0001, by Student's t-test. Data are representative of at least three independent experiments. **Supplemental Fig. 3. (A-B)** Representative flow plot and statistical analysis of SAT ATM and ATDC cells from Lkb1^f/f^ and CD11c^Cre^Lkb1^f/f^ mice with NFD and HFD **(A)** and **(B)** corresponding statistical analysis. **(C-D)** Representative flow plot and statistical analysis of SAT T cell subsets and their effector/memory marker expression **(C)** and **(D)** Statistical analysis the composition of SAT T cells and effector/memory subsets. The results are presented as the mean ± SEM, ^*^P < 0.05, ^**^P < 0.01, ^***^P < 0.001, ^****^P < 0.0001, by Student's t-test. Data are representative of at least three independent experiments. **Supplemental Fig. 4.** Loss of Lkb1 in CD11c^+^ cells limits the accumulation and expression of CD80’s of ATDC but not splenic DCs with classic gating analysis. **(A)** Representative flow plot and statistical analysis of the CD45^+^MHC-II^+^CD11C^hi^ cells in VAT. **(B)** Representative histogram and statistical analysis illustrating CD64^+^ ATMs and CD64^-^ ATDCs from Lkb1^f/f^ and CD11c^Cre^Lkb1^f/f^ mice with NFD and HFD. **(C)** Representative overlay histogram and statistical analysis of the expression of CD80 and CD86 in CD64^-^ATDCs from Lkb1^f/f^ and CD11c^Cre^Lkb1^f/f^ mice with NFD and HFD. **(D)** Representative flow plot and statistical analysis of CD45^+^MHC-II^+^CD11c^hi^ splenic dendritic cells. **(E)** Representative overlay histogram and statistical analysis of the expression of CD80 and CD86 in splenic DCs from Lkb1^f/f^ and CD11c^Cre^Lkb1^f/f^ mice with NFD and HFD. The results are presented as the mean ± SEM, ^*^P < 0.05, ^**^P < 0.01, ^***^P < 0.001, ^****^P < 0.0001, by Student's t-test. Data are representative of at least three independent experiments. **Supplemental Fig. 5**. Gating strategies. **(A)** The gating strategy of CD45^+^ myeloid subsets used in Figure 4 and Figure 6 C. **(B)** The gating strategy of classic DC and ATM used in Supplementary Figure 1. **(C)** The gating strategy of T cells used in Figure 5 and Figure 6 D-E. **(D)** The gating strategy of preadipocytes used in Figure 7. (PDF 629 KB)

## Data Availability

The datasets used and/or analyzed during the current study are available from the corresponding author on reasonable request.
