# Supplementary material for: Loss of Lkb1 in CD11c+ myeloid cells protects mice from diet-induced obesity while enhancing glucose intolerance and IL-17/IFN-γ imbalance
Source: Cell Mol Life Sci. 2023 Feb 13;80(3):63. doi: 10.1007/s00018-023-04707-w (PMC9925521; doi:10.1007/s00018-023-04707-w)

Supplemental figure 1

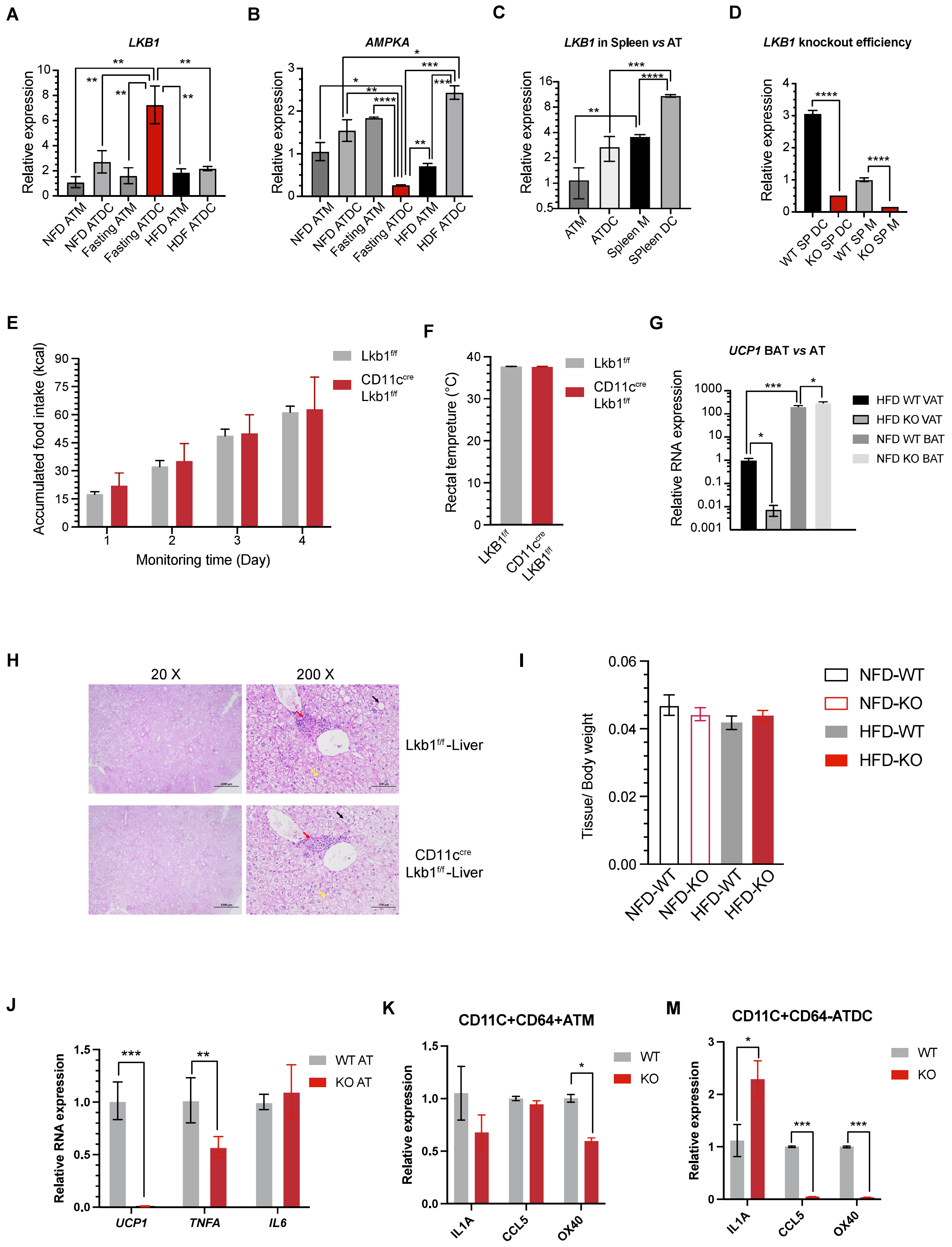

Supplemental figure 2

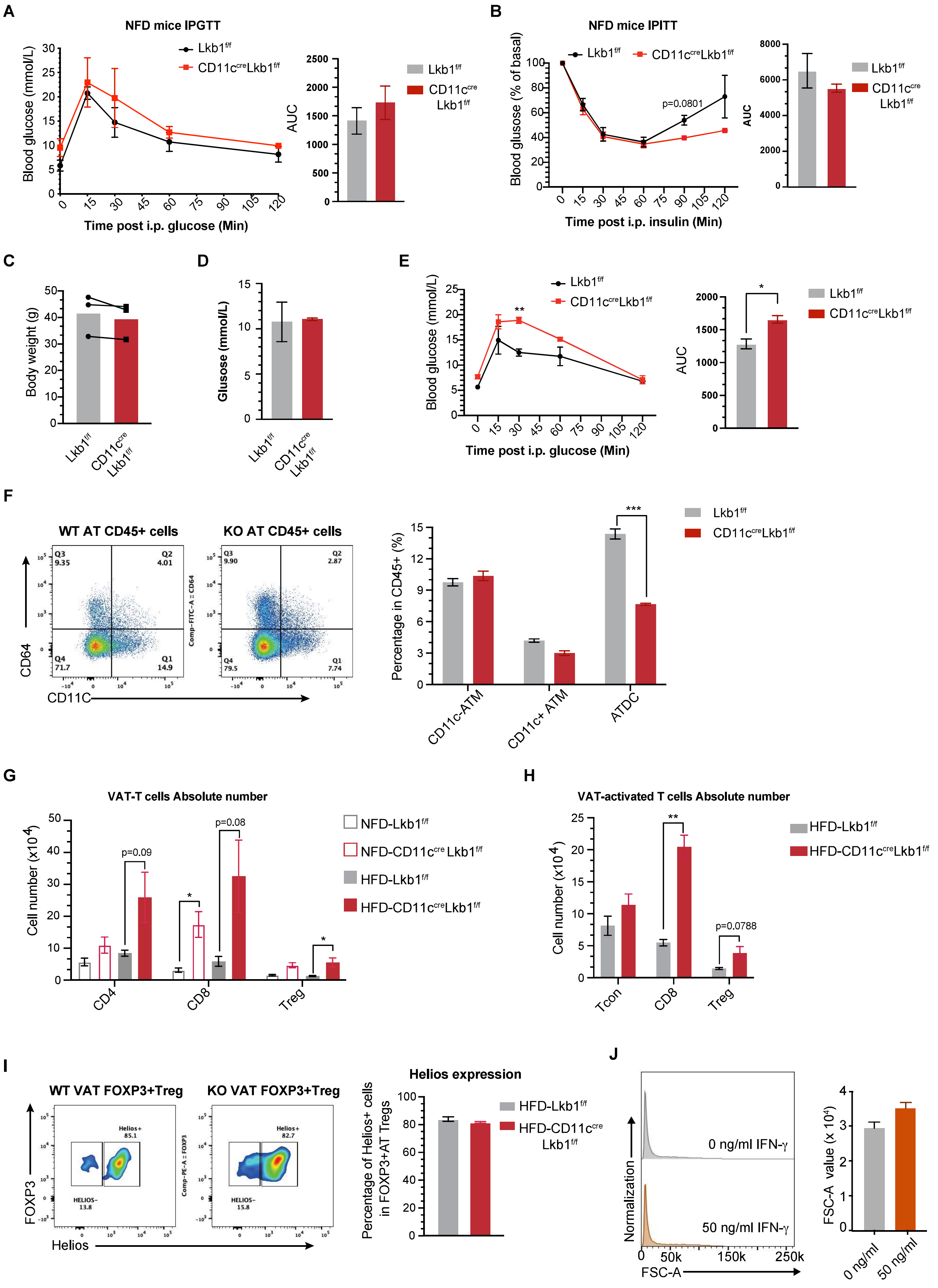

Supplemental figure 3

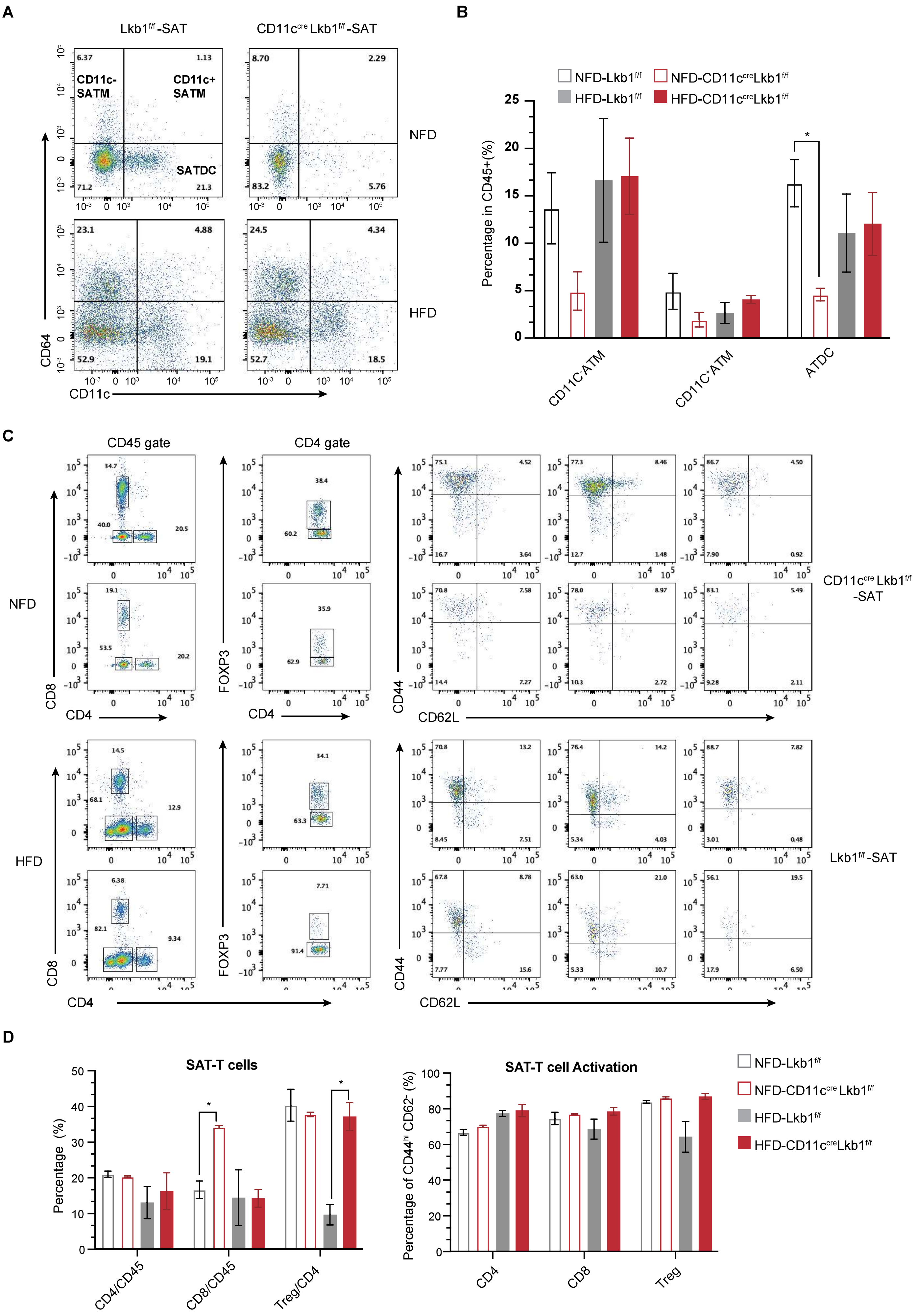

Supplemental figure 4

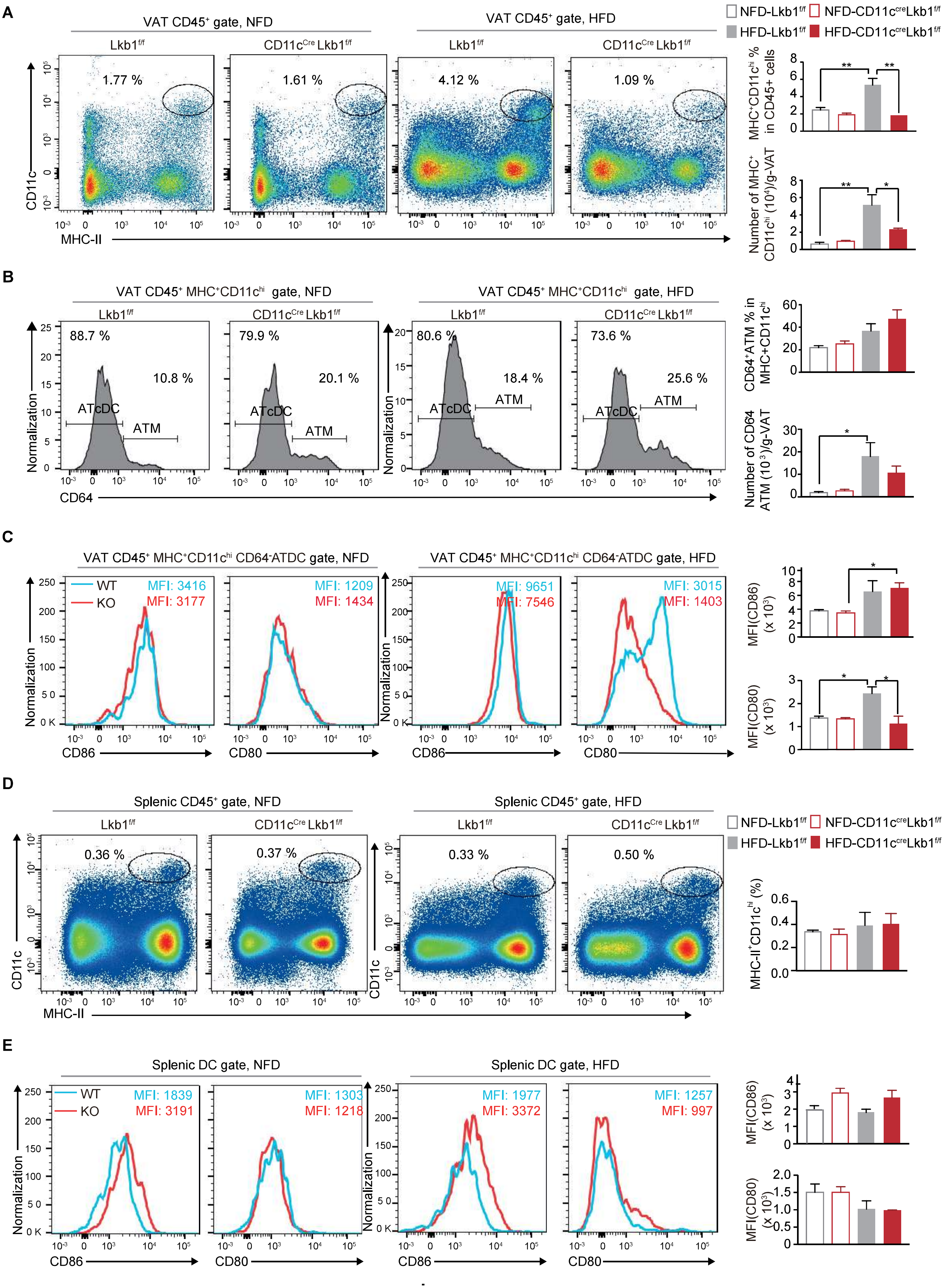

Supplemental figure 5

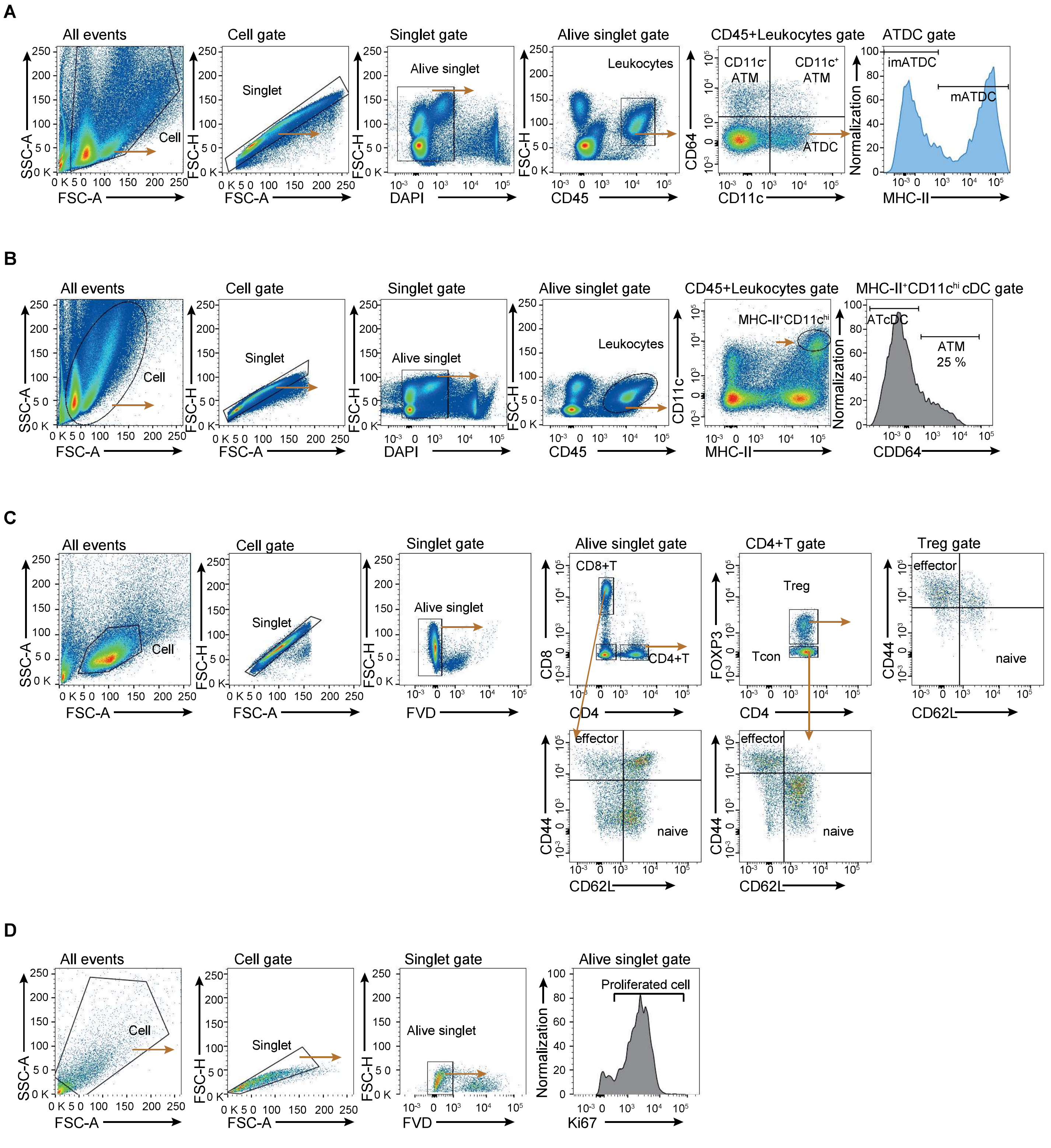

Supplement: Supplementary file 2 — Supplemental Fig. 1. (A-B) mRNA quantitation of LKB1 (A) and AMPKA (B) of adipose tissue CD11c+ cells among fasting, fed, NFD, and HFD conditions. (C) The expression of LKB1 in the adipose tissue and splenic CD11c+ subsets. (D) The expression of LKB1 in splenic CD11c+ subsets from WT and KO mice. (E) Daily monitoring data of accumulated food intake in Lkb1f/f and CD11cCre Lkb1f/f mice fed with HFD (5.24 kcal/g). (F) Monitoring data of rectal temperature in Lkb1f/f and CD11cCre Lkb1f/f mice in DIO model. (G) The UCP1 expression of VAT and BAT tissue from Lkb1f/f mice fed with NFD or HFD. (H) Histological analysis of liver from Lkb1f/f and CD11cCre Lkb1f/f mice in DIO model. The red arrow represents the infiltrated immune cells; the black arrow represents the hepatic steatosis; the yellow arrow represents ballooning degeneration of hepatocytes. (I) Statistical analysis of the liver versus body weight ratio in the indicated groups. (J) The expression of UCP1, TNFA, IL6 in adipose tissue from WT and KO mice. (I) The expression of IL1A, CCL5, OX40 in ATM and ATDC from WT and KO mice. The gating strategy of DC and macrophage are based on the expression of CD64 and CD11c as shown in Supplementary Fig. 5. The cells in A-C were sorted from Lkb1f/f mice. WT and KO represent Lkb1f/f and CD11cCre Lkb1f/f mice, respectively. The results are presented as the mean ± SEM, *P < 0.05, **P < 0.01, ***P < 0.001, ****P < 0.0001, by Student's t-test. Data are representative of at least three independent experiments. Supplemental Fig. 2. (A) Intraperitoneal glucose tolerance test (IPGTT) of mice after 16 weeks of NFD feeding. And statistical analysis of the area under the curve (AUC) of the IPGTT from Lkb1f/f and CD11cCreLkb1f/f mice. (B) Intraperitoneal insulin tolerance test (IPITT) of mice after 16 weeks of NFD feeding. (C-F) Metabolic and immune analysis of the age and body weight matched cohort from Lkb1f/f and CD11cCreLkb1f/f mice with 16 weeks of HFD feeding. (C) Body weight statis [file 18_2023_4707_MOESM2_ESM.pdf]
